# Possible Correlations between the ULF Geomagnetic Signature and Mw6.4 Coastal Earthquake, Albania, on 26 November 2019

**DOI:** 10.3390/e23020233

**Published:** 2021-02-17

**Authors:** Dragoș Armand Stănică, Dumitru Stănică

**Affiliations:** Department of Electromagnetism and Lithosphere Dynamics, Institute of Geodynamics of the Romanian Academy, R-020032 Bucharest, Romania

**Keywords:** ULF geomagnetic signature, Mw6.4earthquake, (PAG)-Bulgaria and Surlari (SUA)-Romania geomagnetic data, BPOL, BPOL*and BPOL* (PAG-SUA) time series

## Abstract

An earthquake of Mw6.4 hit the coastal zone of Albania on 26 November 2019, at 02:54:11 UTC. It was intensively felt at about 34 km away, in Tirana City, where damages and lives lost occurred. To emphasize a pre-seismic geomagnetic signature before the onset of this earthquake, the data collected on the interval 15 October–30 November 2019, at the Panagjurishte (PAG)-Bulgaria and Surlari (SUA)-Romania observatories were analyzed. Further on, for geomagnetic signal identification we used the polarization parameter (BPOL) which is time invariant in non-seismic conditions and it becomes unstable due to the strain effect related to the Mw6.4earthquake. Consequently, BPOL time series and its standard deviations are performed for the both sites using ultra low frequency (ULF)-fast Fourier transform (FFT) band-pass filtering. A statistical analysis, based on a standardized random variable equation, was applied to emphasize on the BPOL* (PAG) and ABS BPOL* (PAG) time series the anomalous signal’s singularity and, to differentiate the transient local anomalies due to the Mw6.4 earthquake, from the internal and external parts of the geomagnetic field, taken PAG observatory as reference. Finally, the ABS BPOL* (PAG-SUA) time series were obtained on the interval 1–30 November 2019, where a geomagnetic signature greater than 2.0, was detected on 23 November and the lead time was 3 days before the onset of the Mw6.4earthquake.

## 1. Introduction

The results carried out using ground-based geomagnetic data and ionospheric perturbations, associated with the catastrophic earthquakes Mw9.0 Tohoku, Japan on March 2011, Mw8.3 Coquimbo, Chile on September 2015 and Mw8.1Chiapas-Mexico, on September 2017 and the Vrancea seismicity, Romania, give useful information to elaborate a specific methodology able to emphasize possible inter-relations between the pre-seismic ultralow frequency (ULF) anomalous geomagnetic signature and the above-mentioned earthquakes [1,2,3,4,5,6,7,8,9,10], taking into account the following three possible earthquake generation mechanisms [11]: (a) piezomagnetic effect [12]; (b) magneto-hydrodynamic effect [13]; (c) Electrokinetic effect [14,15]. As regards the Mw 6.4 earthquake analysis, the following previously contributions at the EGU2020 “Sharing Geosphere Online” are briefly presented further on: a multi parameters analysis of satellite and ground based data (satellite thermal anomalies, atmospheric chemical potential, radon level variation, and very high frequency (VHF) propagation in lower atmosphere) which may supply significant information before an earthquake [16]; a statistical analysis applied to identify a precursory anomaly in the total electron content [17]; observations related to the lower ionospheric turbulence variations in the last half of 2019, in broader Balkan region [18]; INFREP Radio Network revealed variations in connection with six earthquakes (Mw > 5.0) occurred in the Balkan Peninsula and Adriatic Sea on 26 and 27 November 2019 [19]; information regarding the pre-earthquake ionospheric anomalies by using the Romanian VLF/LF Infrep receivers and Gnss Global European Networks [20]; the results carried out by the satellite thermal monitoring of Balkan region by means of robust satellite technique-TIR anomalies in the framework of a multiparametric system are emphasized in [21]. All this information increases our knowledge about the origin of the different pre-seismic signals associated with the above-mentioned catastrophic earthquakes and, subsequently, in this study, the data collected from the two geomagnetic observatories Panagjurishte (PAG), Bulgaria and Surlari (SUA), Romania were analyzed in correlation with Mw6.4earthquake. Further on, to differentiate a pre-seismic anomalous signal associated with this earthquake, a statistical analysis based on the standardized random variable equation, taken observatory PAG as reference, was applied. Finally, it must be mentioned that an anomalous interval, having an apex on 23 November, 2019 on all the following time series of BPOL (PAG), BPOL* (PAG), ABS BPOL* (PAG), and ABS BPOL* (PAG-SUA) was identified, with 3 days before the earthquake occurrence on 26 November, 2019.

## 2. Material and Methods

**Figure 1:**
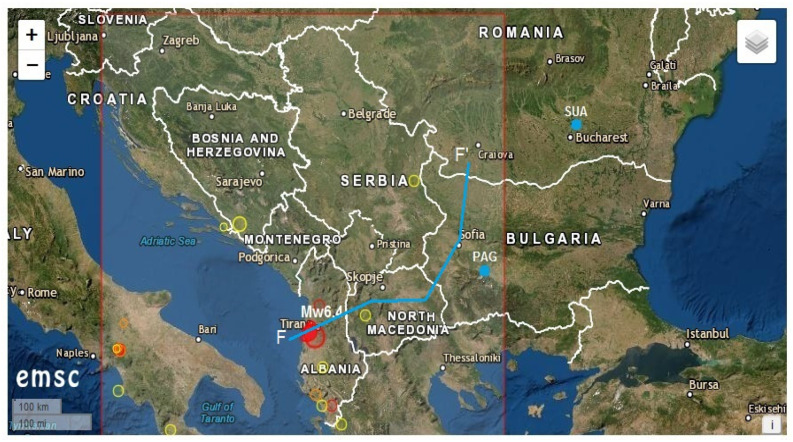
The placements of the Mw6.4 earthquake (red full circle), the geomagnetic observatories Panagjurishte (PAG), Bulgaria and Surlari (SUA), Romania (blue marks) and F-F’ profile (blue line) on the Euro Mediterranean Seismic Centre (EMSC) map.

**Figure 2:**
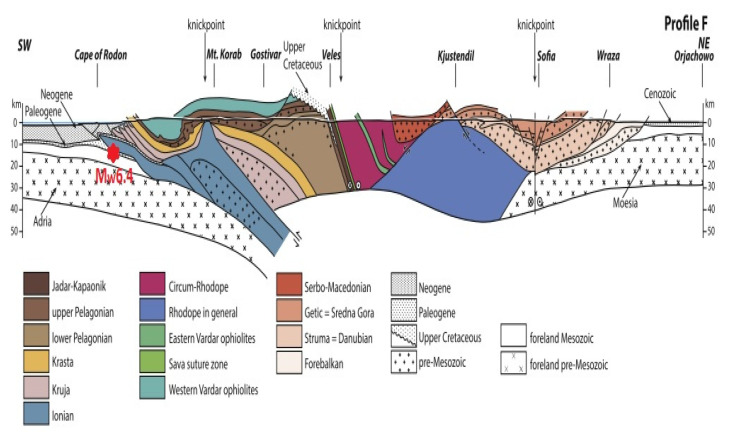
Geotectonic cross-section emphasizing the Mw6.4 earthquake location (red star) on the Adria plate subduction zone, along the profile F –F’ in Figure 1, SW is South-West, NE is North-West, see [22].

A major earthquake of Mw6.4, which was generated at about 10 km depth, hit the coastal zone of Albania on 26 November, 2019 at 02:54:11 UTC, as it was determined by the Euro Mediterranean Seismic Centre (http://www.emsc-csem.org). The main shock was felt in Montenegro, Italy, and Greece (Corfu Island), and it has been followed by more than 100 after-shocks, from which 22 had magnitudes larger than Mw4.0 and four with Mw ≥ 5.0. Both the earthquake epicenter and hypocenter were located near the coastal zone of Albania, at about 30 km distance from the capital city Tirana (Figure 1) and, respectively, on the Adriatic plate subduction zone [22] (Figure 2).

### Basic Theoretical Concepts

To identify pre-seismic geomagnetic signature associated with the Mw6.4earthquake, in this paper, the geomagnetic data were collected, on the interval 15 October–30 November, 2019, via the internet (http://www.intermagnet.org), from the geomagnetic observatories Panagjurishte (PAG), Bulgaria and Surlari (SUA), Romania, and the following relations were used:

(a) Polarization parameter (BPOL) expressed as:

BPOL(f) = Bz(f)/SQRT[(Bx^2^(f) + By^2^(f)] (1)

where Bx, By, and Bz are horizontal and vertical components of the geomagnetic field in µT, f is frequency in Hz [23]. For a given 2D geoelectric structure the vertical magnetic component (Bz) is a totally secondary field being essentially produced by the horizontal magnetic components (Bx, By) and, consequently, BPOL is time invariant in non-seismic conditions that becomes unstable before the onset of the seismic event. In our case, the 2-D structure from the contact between the tectonic units of the Alpine collision zone and the Adria Plate subduction boundary, Figure 2, generates an increased anomalous distribution of BPOL. Its magnitude is proportional with the geoelectric current intensity, due to the tectonic stress generated by the Mw6.4 earthquake

(b) Distance (R) between the observatories and earthquake epicenter is taken into consideration according to the Relation (2), given in [24]

*R* (km) = 10 ^0.5 M−0.27^ (2)

where *R* is epicentral distance and M is earthquake magnitude.

In conformity with Relation (2), the strain effect associated with the Mw6.4 earthquake may be detected up to 800 km. In this case, distances between epicenter and PAG observatory is 450 km and for SUA is about 750 km, respectively, then there are conditions to identify a pre-seismic geomagnetic signature, taking PAG observatory as reference.

**Figure 3:**
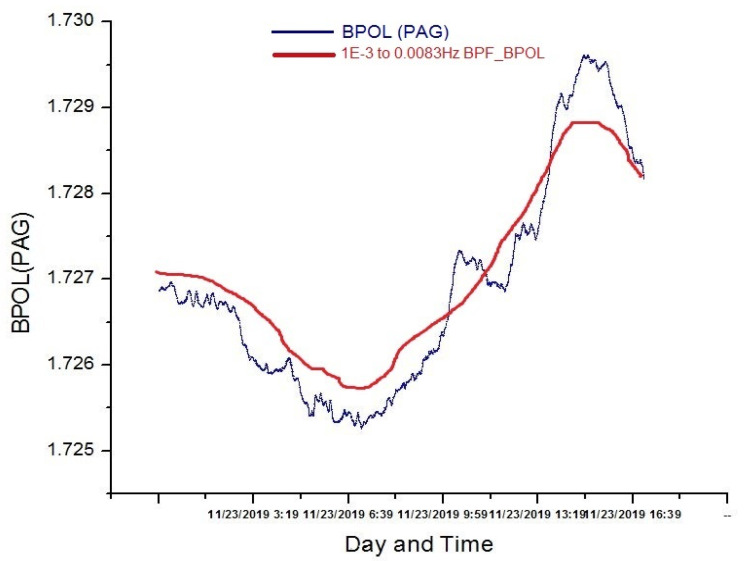
FFT (fast Fourier transform) band-pass filtering (red line) applied on BPOL (PAG) (geomagnetic polarization parameter) distribution for a time window of 1024 samples (∆*t* = 60 s), recorded on 23 November 2019.

The parameters used in this paper in order to identify the precursory geomagnetic anomalies are presented as follows: BPOL (PAG), BPOL (SUA) with their standard deviations (SD), BPOL* (PAG), ABS BPOL* (PAG), and BPOL* (PAG-SUA) are obtained by the next procedures:

FFT-BPF (fast Fourier transform-band pass-filtering) analysis in the ULF (ultra -low frequency) range (0.001–0.0083 Hz) was performed for the two observatories and an example is presented in Figure 3, for PAG observatory 

Statistical analysis based on the standardized random variable equation (Relation 3) to fulfil two objectives:

- to identify geomagnetic precursory signal triggered by Mw6.4 earthquake, that may be observed in the both mentioned observatories, by means of the following relation:

BPOL* = (X − Y)/W(3)

The explanation for X, Y, W may be seen in [8]:

- to separate the precursory seismic signals from the ionospheric and terrestrial geomagnetic field variations, taking PAG observatory as reference, according to the Relation (4):

BPOL* (PAG-SUA) = (A − B)/C(4)

The explanation for the A, B, C, may be also found in [8].

## 3. Results

In this paper, the geomagnetic precursor is considered be generated by the electrical conductivity changes, due to the earthquake generation mechanism that may induce a tectonic stress deployed along the Adria Plate subduction zone (Figure 2). Based on relations (1), (3), (4), in the next three sections (Section 3.1, Section 3.2, Section 3.3), the pre-seismic geomagnetic signatures related to Mw6.4 earthquake are presented.

### 3.1. BPOL (PAG) and BPOL (SUA) Time Series Carried Out Using Relation (1)

**Figure 4:**
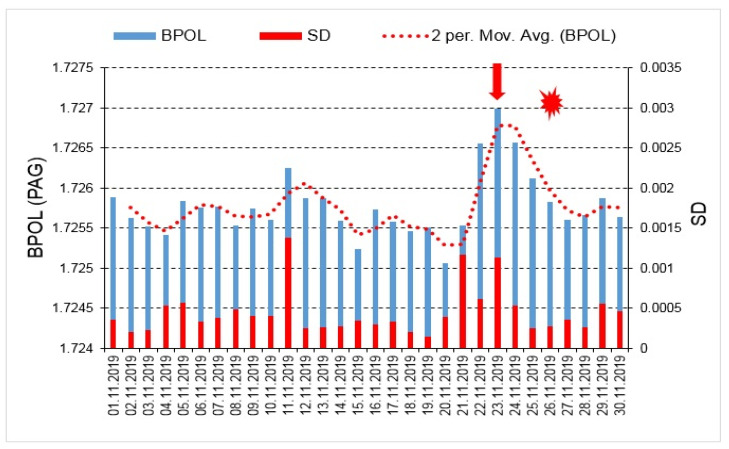
BPOL (PAG) and its standard deviation (SD) time series carried out on the interval 1–30 November 2019; vertical red arrow indicates a pre-seismic anomalous signature on 23 November, 2019; red star is Mw6.4 earthquake; red dotted line is 2 days averaged distribution of BPOL.

**Figure 5:**
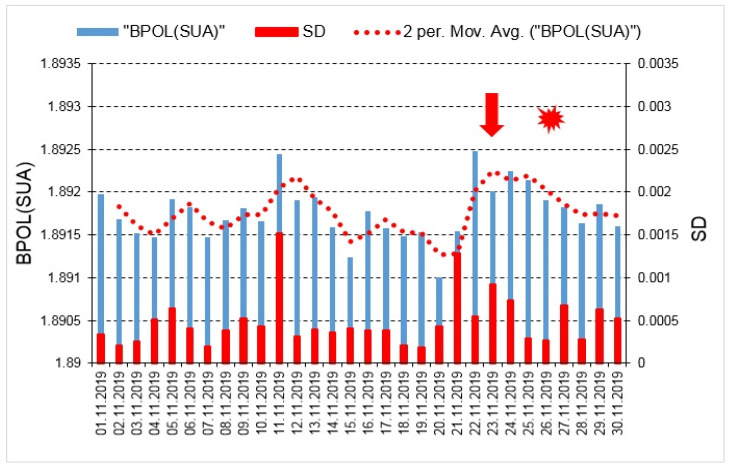
BPOL (SUA) and its standard deviation (SD) time series carried out on the interval 1–30 November 2019; for additional information see Figure 4 caption.

The graphic representations of the BPOL (PAG) and BPOL (SUA) time series led to a comprehensive image on the applied methodology, so as it may be seen in Figure 4 and Figure 5.

### 3.2. BPOL* (PAG) and ABS BPOL* (PAG) Time Series Carried Out Using Relation (3)

**Figure 6:**
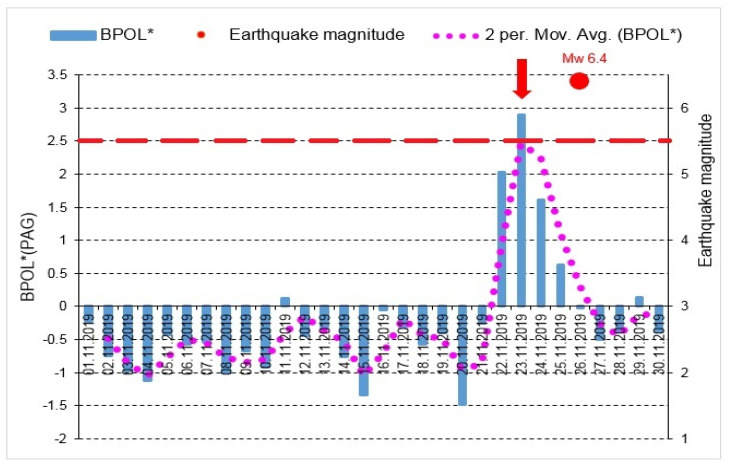
BPOL* (PAG) time series carried out on the interval 1–30 November 2019; vertical red arrow indicates a pre-seismic anomalous signature on 23 November, 2019; red full circle is Mw6.4earthquake; dotted pink line is 2 days averaged distribution of BPOL*; red dashed line is threshold for anomaly using standard deviation (SD).

**Figure 7:**
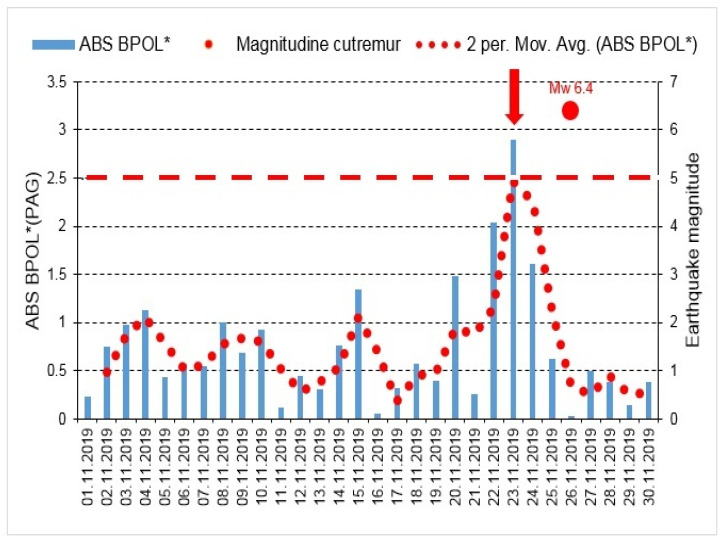
ABS BPOL* (PAG) time series carried out on the interval 1–30 November 2019; ABS is absolute value; for additional explanation see Figure 6 caption.

The graphic models for the BPOL* (PAG), ABS BOPL* (PAG) presented in Figure 6 and Figure 7 put into evidence the precursory geomagnetic anomalies.

### 3.3. ABS BPOL* (PAG-SUA) Time Series Carried Out Using Relation (4)

**Figure 8:**
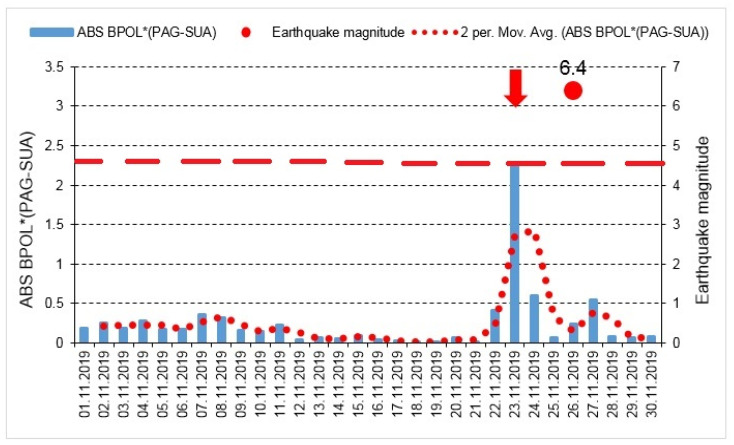
ABS BPOL* (PAG-SUA) time series; for more explanations see Figure 6 and Figure 7 captions.

In the end, in Figure 8, on the time series distribution, it is emphasized an anomalous geomagnetic signal on 23 November, 2019 as a precursor of the Mw6.4, taking as reference PAG observatory.

## 4. Discussion and Conclusions

With the aim to identify possible correlation between the pre-seismic geomagnetic signature and the coastal Mw6.4earthquake, in this paper we investigated the geomagnetic data recorded, on the interval 15 October–30 November, 2019, at the Panagjurishte (PAG), Bulgaria and Surlari (SUA), Romania, the first one taken as reference, and the following results are inferred as:

— The BPOL (PAG) and BPOL (SUA) time series obtained on the interval 1–30 November using (Relation 1), emphasize two pre-seismic anomalous signatures, extended on the intervals 21–27 November for PAG and 21–28 November for SUA, on which two maximum amplitudes were identified on 23 November (1.727 for PAG and 1.892 for SUA), with 3 days before the occurrence of Mw6.4earthquake. These results are presented in Section 3.1, Figure 4 and Figure 5;
— A precursory signature associated to the above-mentioned earthquake was identified on the BPOL* (PAG) and ABS BPOL* (PAG) time series carried out on the interval 1–30 November, using a statistical analysis based on Relation 3, and the results are emphasized in Section 3.2, Figure 6 and Figure 7. On the both BPOL* (PAG) and ABS BPOL* (PAG) time series an anomalous interval, extended between 22 and 24 November, with a maximum value of 2.5 (Figure 6 and Figure 7), was identified with 3 days before the Mw6.4 earthquake occurrence.

To differentiate the transient local anomalies related to the Mw6.4earthquake, by the internal and external parts of the geomagnetic field, we applied Relation 4 to obtain on the interval 1–30 November the ABS BPOL* (PAG-SUA) time series, the geomagnetic observatory (PAG) taken as reference. The result related to the pre-seismic geomagnetic signature, summarized in Section 3.3, Figure 8, consists of a very clear anomaly of maximum extended on 22–24 November, having an apex of about 2.274 on 23 November, identified on the BPOL* (PAG-SUA) time series, with 3 days prior to the onset of the M6.4 earthquake, so as it was indicated by threshold for anomaly (red dashed line).

In conclusion, the above-mentioned results offer opportunities to develop geomagnetic methodologies for the earlier detection of specific pre-seismic anomalies related to the major earthquakes. Consequently, any a priori information related to a major seismic event occurrence, transmitted in time to the authorities responsible in this domain, represents an useful contribution for prevention, management, and decrease of the catastrophic risks.
